# Supplementary material for: Wiz binds active promoters and CTCF-binding sites and is required for normal behaviour in the mouse
Source: eLife. 2016 Jul 13;5:e15082. doi: 10.7554/eLife.15082 (PMC4977153; doi:10.7554/eLife.15082)
Supplement: Figure 2—source data 1. — (A) Nuclear lysates from either pooled E13.5 embryonic brain or 2 adult cerebellum replicates were subjected to antibody-based affinity purification and mass spectroscopy. The number of peptides are shown for each protein identified in both embryonic (values for 1 sample) and adult brain (values for each of the two samples) anti-Wiz antibody IP samples and absent in anti-IgG antibody controls run for each sample, with greater than 5 peptides in each IP. Proteins are ranked by the semi-quantitative number peptides in embryonic brain where Wiz expression is higher. The previously identified Wiz-Zfp644-EHMT1-EHMT2 complex is shaded. DOI: http://dx.doi.org/10.7554/eLife.15082.004 [file elife-15082-fig2-data1.docx]

| Protein identified | # peptides in E13.5 brain IP | # peptides in adult cerebellum IP |
| --- | --- | --- |
| Ehmt2 | [201] | [68,48] |
| Ehmt1 | [161] | [52,41] |
| Whsc1 | [102] | [7,12] |
| Zfp644 | [81] | [24,30] |
| Wiz | [80] | [8,22] |
| Arhgef2 | [45] | [11,15] |
| Nop2 | [41] | [10,12] |
| Strn3 | [30] | [190,122] |
| Zfx | [29] | [21,16] |
| Nifk | [29] | [8,12] |
| Ddx18 | [28] | [6,11] |
| Strn4 | [25] | [43,22] |
| Nvl | [24] | [7,8] |
| Mob4 | [22] | [64,50] |
| Cttnbp2 | [20] | [105,80] |
| No66 | [17] | [8,15] |
| Ahdc1 | [17] | [7,10] |
| Pqbp1 | [16] | [25,9] |
| Rrs1 | [15] | [8,7] |
| Tcof1 | [15] | [8,7] |
| Cep170 | [14] | [8,17] |
| Eef1g | [14] | [9,6] |
| Ighg | [12] | [9,10] |
| Tfip11 | [12] | [6,12] |
| Map7d1 | [11] | [24,24] |
| Spen | [11] | [9,10] |
| Cdc40 | [11] | [13,6] |
| Srp72 | [11] | [10,7] |
| Strn | [10] | [97,64] |
| Ppp2r1a | [9] | [73,55] |
| Ighg2b | [9] | [22,20] |
| Srp68 | [9] | [7,15] |
| Map4 | [9] | [8,9] |
| Rpl35a | [9] | [6,9] |
| Eif6 | [9] | [6,7] |
| Eif2s2 | [8] | [7,7] |
| Kif5b | [7] | [68,24] |
| Strip1 | [7] | [46,13] |
| Pkm | [7] | [12,19] |
| Agap1 | [7] | [18,12] |
| Znf22 | [7] | [8,6] |
| Zfp91 | [6] | [11,6] |
