# Supplementary material for: Wiz binds active promoters and CTCF-binding sites and is required for normal behaviour in the mouse
Source: eLife. 2016 Jul 13;5:e15082. doi: 10.7554/eLife.15082 (PMC4977153; doi:10.7554/eLife.15082)
Supplement: Supplementary file 2. — The average read count from both Wiz+/+and WizMommeD30/+ animals (n = 3 biological replicates per genotype) are shown, with the fold-change (WizMommeD30/+/Wiz+/+) and adjusted significance value for each gene predicted to be differentially expressed. Read counts are normalized for library size. DOI: http://dx.doi.org/10.7554/eLife.15082.015 [file elife-15082-supp2.docx]

| Read count Average wildtype | Read count Average heterozygote | Fold change | Adjusted p-value | Gene ID |
| --- | --- | --- | --- | --- |
| 5119.33 | 2874.72 | 0.56 | 0 | Gm21092 |
| 648.79 | 268.81 | 0.41 | 0 | Rps4l |
| 2439.47 | 1466.30 | 0.60 | 0 | Gm6483 |
| 6493.99 | 4176.69 | 0.64 | 0 | Wiz |
| 1773.20 | 1117.30 | 0.63 | 0 | 6820431F20Rik |
| 2544.85 | 1441.09 | 0.57 | 0 | 2610005L07Rik |
| 995.90 | 571.47 | 0.57 | 0 | Pcdhb17 |
| 601.75 | 346.03 | 0.58 | 0 | Pcdhb20 |
| 440.11 | 231.42 | 0.53 | 0 | Pcdhb18 |
| 666.65 | 365.25 | 0.55 | 0 | Pcdhb16 |
| 295.83 | 153.88 | 0.52 | 0 | AC152164.1 |
| 603.74 | 295.79 | 0.49 | 0 | Gm21769 |
| 749.58 | 404.08 | 0.54 | 0 | Pcdhb19 |
| 360.08 | 221.67 | 0.62 | 0 | Gm10557 |
| 123.82 | 48.59 | 0.39 | 0 | 3222401L13Rik |
| 346.58 | 185.17 | 0.53 | 0 | Sycp1 |
| 2235.19 | 1438.68 | 0.64 | 0 | Pisd-ps1 |
| 159.93 | 82.93 | 0.52 | 0 | Gm26804 |
| 489.64 | 273.48 | 0.56 | 0 | Csf2ra |
| 600.35 | 813.76 | 1.36 | 0 | Ifitm2 |
| 362.75 | 509.59 | 1.40 | 0 | Slc43a3 |
| 467.12 | 301.74 | 0.65 | 0 | Pcdhb22 |
| 167.85 | 93.69 | 0.56 | 0 | Pcdhb15 |
| 149.66 | 78.34 | 0.52 | 0 | Pcdhb14 |
| 187.61 | 116.88 | 0.62 | 0 | Pcdhb10 |
| 285.47 | 193.28 | 0.68 | 0 | Pcdhb5 |
| 128.79 | 69.00 | 0.54 | 0 | Pcdhb12 |
| 249.94 | 172.76 | 0.69 | 0.01 | Pcdhb11 |
| 168.19 | 102.52 | 0.61 | 0.01 | Gm21811 |
| 241.27 | 165.29 | 0.69 | 0.01 | Samd11 |
| 119.62 | 67.28 | 0.56 | 0.01 | Pcdhb13 |
| 577.44 | 776.13 | 1.34 | 0.01 | Calb1 |
| 74.72 | 131.52 | 1.76 | 0.02 | Gm14418 |
| 333.93 | 200.89 | 0.60 | 0.03 | Gm21967 |
| 229.26 | 147.01 | 0.64 | 0.04 | Mmp9 |
| 520.55 | 774.37 | 1.49 | 0.04 | Heph |
