# Supplementary material for: Wiz binds active promoters and CTCF-binding sites and is required for normal behaviour in the mouse
Source: eLife. 2016 Jul 13;5:e15082. doi: 10.7554/eLife.15082 (PMC4977153; doi:10.7554/eLife.15082)
Supplement: Supplementary file 3. — The average read count from both Wiz+/+ and WizMommeD30/+ animals (n = 2 biological replicates per genotype) are shown, with the fold-change (WizMommeD30/+/Wiz+/+) and adjusted significance value for each gene predicted to be differentially expressed. Read counts are normalized for library size. DOI: http://dx.doi.org/10.7554/eLife.15082.016 [file elife-15082-supp3.docx]

| Read count Average wildtype | Read count Average heterozygote | Fold change | Adjusted p-value | Gene ID |
| --- | --- | --- | --- | --- |
| 996.91 | 324.61 | 0.33 | 0 | Rps4l |
| 6645.79 | 3495.44 | 0.53 | 0 | Gm21092 |
| 3402.83 | 1741.12 | 0.51 | 0 | 2610005L07Rik |
| 2959.99 | 1607.94 | 0.54 | 0 | Gm6483 |
| 767.36 | 322.41 | 0.42 | 0 | Gm21769 |
| 2310.29 | 1250.36 | 0.54 | 0 | 6820431F20Rik |
| 1476.22 | 746.58 | 0.51 | 0 | Pcdhb17 |
| 453.11 | 171.85 | 0.38 | 0 | Sycp1 |
| 909.97 | 457.45 | 0.50 | 0 | Pcdhb16 |
| 406.94 | 161.03 | 0.40 | 0 | AC152164.1 |
| 619.83 | 289.11 | 0.47 | 0 | Pcdhb18 |
| 8734.76 | 5867.64 | 0.67 | 0 | Wiz |
| 764.18 | 415.26 | 0.54 | 0 | Csf2ra |
| 289.90 | 104.82 | 0.36 | 0 | Pcdhb14 |
| 581.56 | 311.49 | 0.54 | 0 | Gm26853 |
| 752.10 | 426.22 | 0.57 | 0 | Pcdhb22 |
| 171.07 | 56.60 | 0.33 | 0 | 3222401L13Rik |
| 265.23 | 108.78 | 0.41 | 0 | Pcdhb15 |
| 454.95 | 238.58 | 0.52 | 0 | Pcdhb11 |
| 593.26 | 337.79 | 0.57 | 0 | Pcdhb21 |
| 385.32 | 199.21 | 0.52 | 0 | Pcdhb6 |
| 197.70 | 79.00 | 0.40 | 0 | Gm26804 |
| 3558.39 | 2147.38 | 0.60 | 0 | Pisd-ps1 |
| 336.22 | 173.33 | 0.52 | 0 | Pcdhb10 |
| 923.51 | 473.28 | 0.51 | 0 | Pcdhb20 |
| 457.01 | 260.85 | 0.57 | 0 | Gm10557 |
| 201.10 | 95.33 | 0.47 | 0 | Pcdhb13 |
| 544.04 | 339.77 | 0.62 | 0 | Pcdhb3 |
| 183.60 | 86.43 | 0.47 | 0 | Lipo2 |
| 258.11 | 138.04 | 0.53 | 0 | Pcdhb2 |
| 214.01 | 106.78 | 0.50 | 0.01 | Pcdhb12 |
| 219.89 | 117.76 | 0.54 | 0.01 | Gm21811 |
| 337.20 | 201.72 | 0.60 | 0.01 | Mmp9 |
| 288.68 | 166.93 | 0.58 | 0.01 | Otof |
| 1076.49 | 514.88 | 0.48 | 0.01 | Pcdhb19 |
| 321.97 | 193.84 | 0.60 | 0.01 | Fgf3 |
| 861.24 | 607.50 | 0.71 | 0.01 | Gm26672 |
| 763.76 | 515.97 | 0.68 | 0.02 | Stag3 |
| 650.72 | 447.17 | 0.69 | 0.02 | Cybrd1 |
| 300.31 | 138.45 | 0.46 | 0.02 | Nlrp5-ps |
| 799.87 | 564.64 | 0.71 | 0.03 | Pisd-ps2 |
| 256.32 | 151.08 | 0.59 | 0.03 | Pcdhb4 |
| 527.95 | 349.04 | 0.66 | 0.03 | Olig1 |
| 7.07 | 36.80 | 5.21 | 0.03 | S100a8 |
