# Supplementary material for: Wiz binds active promoters and CTCF-binding sites and is required for normal behaviour in the mouse
Source: eLife. 2016 Jul 13;5:e15082. doi: 10.7554/eLife.15082 (PMC4977153; doi:10.7554/eLife.15082)
Supplement: Supplementary file 4. — The average read count from both Wiz+/+ and WizMommeD30/+ animals (n = 3 biological replicates per genotype) are shown, with the fold-change (WizMommeD30/+ / Wiz+/+) and adjusted significance value for each gene predicted to be differentially expressed. Cells highlighted orange are protocadherin β cluster genes and cells highlighted green are cadherin 11-like cluster genes. Read counts are normalized for library size. DOI: http://dx.doi.org/10.7554/eLife.15082.017 [file elife-15082-supp4.docx]

| Read count Average wildtype | Read count Average heterozygote | Fold-change | Adjusted  p-value | Gene ID |
| --- | --- | --- | --- | --- |
| 21.94331 | 4.991446 | 0.22747 | 0.003551 | Slc6a2 |
| 44.80236 | 17.2675 | 0.385415 | 0.008374 | Gm3912 |
| 38.08069 | 15.89144 | 0.41731 | 0.013056 | Vmn2r2 |
| 161.7897 | 78.24647 | 0.483631 | 1.25E-08 | Gm21769 |
| 157.3716 | 76.11884 | 0.483689 | 2.16E-05 | Rps4l |
| 52.81149 | 27.1712 | 0.514494 | 0.026213 | Gcgr |
| 78.801 | 41.3091 | 0.524221 | 0.003315 | AC152164.1 |
| 55.69326 | 29.94845 | 0.537739 | 0.032009 | Rnaset2a |
| 799.8781 | 431.2873 | 0.539191 | 0.030098 | Zbtb16 |
| 145.8374 | 85.50059 | 0.586273 | 0.041323 | Myo5b |
| 99.47894 | 62.58478 | 0.629126 | 0.025582 | Gm21811 |
| 356.0909 | 225.6939 | 0.63381 | 2.03E-06 | Pcdhb14 |
| 286.0199 | 182.0906 | 0.636636 | 0.032596 | Trpv4 |
| 3154.761 | 2044.397 | 0.648036 | 3.47E-42 | Gm21092 |
| 221.7414 | 144.6031 | 0.652125 | 0.007859 | Chil5 |
| 1460.975 | 955.6826 | 0.65414 | 6.4E-22 | Gm6483 |
| 1214.56 | 798.1862 | 0.657181 | 4.98E-21 | 6820431F20Rik |
| 324.9392 | 215.2613 | 0.662466 | 0.037732 | Scand1 |
| 2614.977 | 1748.625 | 0.668696 | 5.49E-11 | 2610005L07Rik |
| 123.5592 | 82.67271 | 0.669094 | 0.029495 | Pcdhb2 |
| 129.3811 | 86.80279 | 0.670908 | 0.027218 | 3222401L13Rik |
| 143.5468 | 96.60735 | 0.673002 | 0.017917 | Gm26853 |
| 140.5133 | 94.8426 | 0.674973 | 0.021125 | Samd11 |
| 218.2958 | 148.0847 | 0.678367 | 0.006929 | Csf2ra |
| 258.9647 | 177.7322 | 0.686318 | 0.006948 | Pcdhb3 |
| 171.2655 | 119.0998 | 0.69541 | 0.015657 | Cpne6 |
| 138.1147 | 96.27099 | 0.697037 | 0.043216 | Agpat9 |
| 361.4466 | 252.6605 | 0.699026 | 0.021048 | Hspa12b |
| 2845.939 | 2010.554 | 0.706464 | 1.49E-25 | Wiz |
| 1608.911 | 2096.475 | 1.30304 | 4.19E-12 | Arhgap20 |
| 289.6806 | 378.1217 | 1.305305 | 0.006221 | Il18 |
| 522.5754 | 682.9409 | 1.306875 | 0.000219 | Nfkbia |
| 966.4311 | 1266.349 | 1.310335 | 8.22E-09 | Trp53i11 |
| 185.7364 | 244.8147 | 1.318076 | 0.036078 | Sult1a1 |
| 9281.764 | 12268.71 | 1.321808 | 3.67E-16 | Nos1 |
| 307.3035 | 406.5146 | 1.322844 | 0.019056 | Ldoc1 |
| 225.0103 | 298.9945 | 1.328803 | 0.017554 | Zfp57 |
| 310.5083 | 414.9572 | 1.33638 | 0.00101 | Dmp1 |
| 259.4407 | 347.0519 | 1.337693 | 0.003554 | Xkr4 |
| 276.4359 | 370.7041 | 1.341013 | 0.00188 | Dhdh |
| 266.9072 | 358.9262 | 1.34476 | 0.002088 | Tspan18 |
| 196.2509 | 264.8302 | 1.349447 | 0.011289 | Frmpd3 |
| 272.6451 | 369.7639 | 1.35621 | 0.001004 | Ptar1 |
| 135.9642 | 186.6351 | 1.372678 | 0.039216 | Kirrel2 |
| 326.1855 | 451.2725 | 1.383484 | 3.78E-05 | Bcl2l11 |
| 388.0835 | 546.0171 | 1.406958 | 5.7E-07 | Col12a1 |
| 148.4508 | 209.2164 | 1.409331 | 0.010336 | Gm13152 |
| 127.8427 | 180.3707 | 1.41088 | 0.021243 | Hmgcs2 |
| 485.0451 | 684.4241 | 1.411052 | 0.000996 | Etnppl |
| 126.3428 | 178.4163 | 1.412161 | 0.020584 | Col4a6 |
| 119.7199 | 170.2599 | 1.422153 | 0.021125 | Il22 |
| 371.2775 | 530.2062 | 1.428059 | 2.42E-07 | Slc5a1 |
| 1168.904 | 1687.56 | 1.443712 | 0.005578 | Lnpep |
| 676.2746 | 981.1005 | 1.450743 | 2.44E-07 | Adamts18 |
| 388.4125 | 566.7937 | 1.459257 | 1.31E-08 | Ddr2 |
| 218.8104 | 322.4796 | 1.473785 | 0.01518 | Slco2a1 |
| 107.9745 | 160.0194 | 1.482011 | 0.009573 | Gbp6 |
| 176.7156 | 262 | 1.482608 | 0.023577 | Fmo2 |
| 244.8861 | 366.3536 | 1.496016 | 1.37E-06 | Bmp5 |
| 343.3702 | 514.6384 | 1.498786 | 4.53E-09 | Abi3bp |
| 105.0948 | 158.2233 | 1.505529 | 0.01225 | Mex3a |
| 206.4847 | 311.7102 | 1.509604 | 9.83E-06 | Chrdl1 |
| 134.0497 | 202.7305 | 1.512354 | 0.001004 | Cd74 |
| 409.218 | 622.1546 | 1.52035 | 7.54E-12 | Alpk2 |
| 189.5575 | 288.3731 | 1.521296 | 1.3E-05 | Casp12 |
| 293.888 | 447.2352 | 1.521788 | 0.003877 | 1700048O20Rik |
| 70.91932 | 109.8432 | 1.548848 | 0.025036 | Galr1 |
| 98.80859 | 153.282 | 1.551302 | 0.003551 | Fam198b |
| 313.3152 | 493.4191 | 1.574833 | 4.61E-11 | Tdo2 |
| 72.96621 | 115.2805 | 1.579916 | 0.013027 | Ano5 |
| 200.076 | 319.2303 | 1.595545 | 0.045642 | Acer2 |
| 162.3114 | 262.7729 | 1.618943 | 1.33E-06 | A930017K11Rik |
| 183.6101 | 299.5775 | 1.631596 | 7.3E-08 | Tfpi |
| 40.36274 | 68.39885 | 1.694604 | 0.044578 | Gm16503 |
| 45.03455 | 76.92582 | 1.708151 | 0.022149 | Magix |
| 86.9996 | 148.9501 | 1.712078 | 0.000178 | Cdc20 |
| 300.6732 | 542.0262 | 1.802709 | 5.38E-05 | Gm15083 |
| 42.22665 | 81.14744 | 1.921711 | 0.002082 | Vit |
| 42.45298 | 81.61737 | 1.922536 | 0.037031 | Apoc1 |
| 77.16693 | 161.3968 | 2.091528 | 5.2E-09 | Arg1 |
| 64.01607 | 158.6927 | 2.478952 | 9.63E-13 | Casp1 |
| 18.31883 | 48.21622 | 2.632058 | 0.000769 | Olfm4 |
