# Supplementary material for: Wiz binds active promoters and CTCF-binding sites and is required for normal behaviour in the mouse
Source: eLife. 2016 Jul 13;5:e15082. doi: 10.7554/eLife.15082 (PMC4977153; doi:10.7554/eLife.15082)
Supplement: Supplementary file 5. — DOI: http://dx.doi.org/10.7554/eLife.15082.018 [file elife-15082-supp5.docx]

| Primer name | 5' to 3' |
| --- | --- |
| Agouti_3’_GenotypeR | TGGCCAGGAAAGAAGGAAACTG |
| Agouti_5’_GenotypeF | CATGGCTACAGCATCCTGACAA |
| IAP_5’_GenotypeF | GCGCATCACTCCCTGATTG |
| Wiz_GenotypeF | AATGGCTCTCCCATTGACAC |
| Wiz_GenotypeR | GGGCAGTTCAGTCTGGATGT |
| Ychr_YNLS.5 | CCTATTGCATGGACAGCAGCTTATG |
| Ychr_Zfyl.8b | GACTAGACATGTCTTAACATCTGTCC |
| Xchr_OTCFOR | GTTCTTTCGTTTTCCCCTCTC |
| Xchr_OTCREV | GGCATTATCTAAGGAGAAGCATC |
